# Lower odds of COVID-19-related mortality in hospitalised patients with type II diabetes mellitus: A single-centre study

**DOI:** 10.1371/journal.pone.0287968

**Published:** 2023-11-17

**Authors:** Jonathan Mina, Nadia L. Samaha, Mohamad Fleifel, Janane Nasr, Tony Haykal, Hani Dimassi, Ranime Harb, Ghida El Hout, Elissar Franjieh, Ahmad Mahdi, Jacques Mokhbat, Anna Farra, Rola Husni

**Affiliations:** 1 Department of Internal Medicine, Staten Island University Hospital, Staten Island, New York, United States of America; 2 Georgetown University School of Medicine, Washington, D.C., United States of America; 3 Department of Internal Medicine, Lebanese American University Medical Centre-Rizk Hospital, Beirut, Lebanon; 4 Gilbert and Rose-Marie Chagoury School of Medicine, Lebanese American University, Byblos, Lebanon; 5 School of Pharmacy, Lebanese American University, Byblos, Lebanon; Tabba Heart Institute, PAKISTAN

## Abstract

**Background:**

COVID-19 infection in patients with type 2 diabetes mellitus (T2DM) -a chronic illness in Lebanon–is not well described.

**Methods:**

This was a single-centre retrospective observational study of 491 patients, including 152 patients with T2DM, who were hospitalised for COVID-19 between 20 August 2020 and 21 April 2021. Data on clinical characteristics, laboratory and radiological findings and outcomes were collected from the electronic medical records. Clinical characteristics and in-hospital mortality between patients with and without T2DM infected with COVID-19 using multivariate analysis were compared.

**Results:**

Patients with T2DM were significantly older than those without T2DM (mean age, 68.7 vs. 60.3 years). Patients with T2DM were more likely to present with a body temperature of <38.3°C (83.9% vs. 69.9%) and less likely to present with chest pain (3.9% vs. 9.1%) and sore throat (2.0% vs. 6.8%). Patients with T2DM were more likely to be hypertensive (76.35% vs. 41%) and dyslipidaemic (58.6% vs. 25.7%) and had more frequent underlying coronary artery disease (33.6% vs. 12.4%). The rates of patients with creatinine levels of ≥1.17 mg/L and troponin T levels of ≥4 ng/dL were higher in the T2DM group than in the non-T2DM group (30.4% vs. 15% and 93.3% vs. 83.1%, respectively). Patients with T2DM were more likely to be admitted to the intensive care unit (ICU) (34.2% vs. 22.1%), require invasive ventilation (18.4% vs. 10.3%) and receive vasopressors (16.4% vs. 10.0%). Increasing age and the use of invasive ventilation and vasopressors were associated with higher odds of mortality (odds ratio (OR), 1.08, 9.95 and 19.83, respectively), whereas longer ICU stay was associated with lower odds of mortality (OR, 0.38). The odds of mortality were lower in the T2DM group than in the non-T2DM group (OR, 0.27).

**Conclusion:**

Among patients hospitalised for COVID-19, those with T2DM were older, presented with milder symptoms and had more comorbidities and higher troponin T levels compared with those without T2DM. Despite the worse clinical course, the patients with T2DM had lower odds of mortality than those without T2DM.

## Introduction

The coronavirus disease 2019 (COVID-19) pandemic has ravaged the world, affecting over 561 million people worldwide and leading to over 6.35 million deaths to date [1]. COVID-19 not only has gravely threatened the global health but also has led to the collapse of many economies, with an estimated cost of 16 trillion US dollars worldwide [2].

COVID-19 ranges from mild self-limiting illness to pneumonia, respiratory failure and death. Since the start of the COVID-19 pandemic, a plethora of studies have investigated the pathogenesis, virulence and mode of transmission of the severe acute respiratory syndrome (SARS) coronavirus 2 (SARS-CoV-2) as well as the manifestations of COVID-19 in different patient populations. Most studies to date have demonstrated that the clinical COVID-19 outcomes are worse in patients with certain comorbidities such as hypertension, type 2 diabetes mellitus (T2DM) and heart disease [3]. Importantly, in past respiratory disease outbreaks such as severe acute respiratory syndrome (SARS) and the Middle East respiratory syndrome (MERS), DM has been consistently shown to increase the risk of morbidity and mortality related to acute infections due to suppressed innate and humoral immune function [4, p19]. Akbar et al. reported that glycated haemoglobin above 9% was associated with a 60% increase in hospitalisation risk and pneumonia severity in patients with pneumonia [5]. During the 2002–2003 outbreak of SARS-CoV-1, high plasma glucose levels and T2DM were risk factors for morbidity and mortality in patients with SARS [6]. Similarly, during the 2012 outbreak of Middle East Respiratory Syndrome coronavirus, epidemiological studies demonstrated that approximately 50% of the patient population had DM and that the odds ratio (OR) for severe/critical Middle East Respiratory Syndrome coronavirus infection was between 7.2 and 15.7 in patients with DM compared with the overall population [7].

Expectedly, with the progression of the COVID-19 pandemic, patients with T2DM have been identified as one of the critical populations with worse COVID-19 outcomes. The majority of studies to date provide ample evidence linking T2DM with increased risk of hospitalisation, metabolic emergencies, mechanical ventilation and intensive care unit (ICU) admission following COVID-19 infection [8,9]. Additionally, T2DM has been shown to be strongly associated with increased incidence and severity of COVID-19 [4,10]. Multiple studies categorise T2DM as a risk factor for in-hospital mortality, in addition to other risk factors, primarily age >50 years, male sex, immunosuppression and other comorbidities such as renal, lung and cardiovascular diseases [11]. The impact of DM on certain parameters such as ICU admission and disease severity has been investigated, but its effect on mortality and outcomes of hospitalised patients with COVID-19 remains contested. One French study that aimed to determine whether T2DM negatively impacted death rates in COVID-19 patients reported that T2DM in fact did not impact mortality, although it was associated with higher ICU admission rate and protracted hospital stay compared with that observed in patients without T2DM [12]. These findings highlight the need for further investigation as the relationship between T2DM and COVID-19 is an intricate, nuanced relationship complicated by multiple factors. Patients with T2DM tend to be older than the general population and often present with comorbidities such as hypertension, obesity, cardiovascular disease and renal disease, which can independently change clinical outcomes and affect mortality rates in their own right.

Similar population-based studies examining the relationship between T2DM and COVID-19 differ in ethnic background, genetics and disease prevalence, preventing the extrapolation of data from one country to another [13]. An estimated 6.28% of the global population is affected by T2DM [14]. In 2019, T2DM was the ninth leading cause of death worldwide, leading to over 1.5 million deaths directly caused by T2DM [15]. In Lebanon, the prevalence of T2DM among adults is even higher, affecting 8.9% of the total population [16]. Therefore, T2DM is a major comorbidity to consider in patients with COVID-19 in Lebanon. To date, no study in Lebanon has examined the impact of COVID-19 on disease outcomes in patients with T2DM. The present study aimed to uncover the prevalent comorbidities and the clinical course and outcomes of COVID-19 following hospitalisation in patients with T2DM in comparison with those without T2DM.

## Methods

This was a single-centre retrospectively observational chart review study of adult 491 patients who were hospitalised for COVID-19 after confirming infection with RT-PCR in Lebanese American University Medical Centre-Rizk Hospital between 20 August 20 2020 and 21 April 2021. Of note, the alpha and delta variants were the most common circulating SARS-CoV-2 strains during the study period. Patients aged younger than 18 years were excluded. In addition, patients who were admitted for less than 24 h were excluded because of the lack of data detailing the disease course. Of the 491 patients in the study, 152 were previously diagnosed with T2DM. Data were collected from the electronic medical records and included demographics (age, sex and smoking status), signs and symptoms at presentation (fever, chills, cough, dyspnoea, desaturation, diarrhoea, abdominal pain, nausea, vomiting, myalgia, chest pain and sore throat), comorbidities, length of hospitalisation, admission to ICU (at presentation or later during the hospital stay if needed), ICU length of stay, use of invasive ventilation and vasopressors, radiological findings (chest X-ray and computed tomography [CT]), laboratory results, treatments (antiviral, corticosteroids, anticoagulation, vitamin C, zinc and antibiotics), plasma transfusion and outcome statuses being either in-hosptial mortality or improvement and discharge.).

This study was reviewed and approved on April 2^nd^ 2020 by the Lebanese American University (LAU) Institutional Review Board (IRB) with the reference number LAUMCRH.RH2.2/Apr/2020.

### Ethical statement approval

All patient data was anonymized before access in this retrospective chart review. The LAU IRB provided approval to access the patient records. Patients did not provide written informed consent as per study design and IRB approval.

### Statistical analysis

The Pearson chi-square test was used to compare patients with and without T2DM. The variables extracted from the medical charts included general patient characteristics, symptoms, medical history, laboratory results and clinical outcomes. Multivariate logistic regression analysis was used with the outcome of hospitalisation as the dependent variable, after removing patients with unknown definitive outcomes such as transfer to other hospitals. All independent variables with a p value of <0.20 with the dependent variable at the bivariate level were included in the model. Coefficients and standard errors were exponentiated to calculate ORs with 95% confidence intervals (CIs).*Post hoc* power analysis was performed (OR for a major outcome, 0.27; alpha, 0.05; the proportion of outcome among exposure, 15%; the proportion of T2DM, 30%; and R2 for all other covariates set between 40% and 30%), with our calculated power is 83% and up to 88% confirming we have enough power for the multivariate logistic regression. For all analyses, a p value of 0.05 was considered to indicate statistical significance. All statistical analyses were conducted using SPSS.

## Results

As shown in **Table 1**, patients previously diagnosed with T2DM comprised 31% (152 patients) of the total study cohort of 491 patients. The mean age of the patients with T2DM was significantly higher than that of the patients without T2DM (68.73 vs. 60.37 years, p < 0.001). Additionally, the percentage of patients with T2DM between 65 and 74 years of age was 32.9%, equal to the percentage of patients with T2DM 75 years of age and older (32.9%). In comparison, 19.8% and 22.4% of the patients without T2DM were between 65 and 74 years of age and 75 years of age and older, respectively. In the overall cohort, the patients hospitalised for COVID-19 were predominantly male. Moreover, 71.7% of the patients with T2DM were male, compared with 67.6% male proportion in patients without T2DM, without a statistically significant difference. Finally, smoking status was not significantly different between the two groups.

**Table 1:** Demographical characteristics of diabetic and non-diabetic patients admitted for COVID-19 infection.

	Type 2 Diabetes		No Type 2 Diabetes		
	N(%)		N(%)		*p*-value
**Total Sample**	152(31.0%)		339 (69.0**%)**		
**Demographics**					
**Age: mean ± stdev**	68.73(12.518)		60.37 (16.431%)	16.431	
Age 17 to 44	6(3.9%)		60 (17.7%)	17.7**%**	
Ages 45 to 64	46(30.3%)		136 (40.1%)	40.1**%**	
Ages 65 to 74	50(32.9%)		67 (19.8%)	19.8**%**	
Ages 75 and above	50(32.9%)		76 (22.4%)	22.4**%**	<0.001
**Sex**					
Males	109(71.7%)		229 (67.6%)	67.6**%**	
Females	43(28.3%)		110 (32.4%)	32.4**%**	0.358
**Smoking status**					
Never-smoker	114(79.2%)		240 (75.2%)	75.2**%**	
Ex-smoker	5(3.5%)		8 (2.5%)	2.5**%**	
Smoker	25(17.4%)		71 (22.3%)	22.3**%**	0.433

**Table 2** shows the comparison of the signs and symptoms related to COVID-19 at admission. The rate of patients with a body temperature below 38.3°C was significantly higher in the T2DM group than in the non-T2DM group (83.9% vs. 69.9%, p < 0.005). Significantly fewer patients with T2DM reported chest pain and sore throat compared with those without T2DM (3.9% vs. 9.1%, p = 0.044 and 2.0% vs. 6.8%, p = 0.028, respectively). The most common symptoms of the patients with T2DM were dyspnoea (65.8%), fever (64.5%), cough (42.1%) and myalgia (32.2%). No statistically significant differences in other clinical signs and symptoms, including chills, cough, dyspnoea, myalgia and gastrointestinal symptoms were noted between the two groups.

**Table 2:** Signs and symptoms of diabetic and non-diabetic patients admitted for COVID-19 infection.

	Type 2 Diabetes		No Type 2 Diabetes		
	N(%)		N(%)		*p*-value
**Chief Complaints**					
Fever	98 (64.5%)	64.5**%**	236 (69.6%)	69.6**%**	0.259
Temperature range					0.005
*<38.3°C*	125 (83.9%)	83.9**%**	235 (69.9%)	69.9**%**	
*38.3°C– 39.3°C*	20 (13.4%)	13.4**%**	86 (25.6%)	25.6**%**	
*≥39.3°C*	4 (2.7%)	2.7**%**	15 (4.5%)	4.5**%**	
Chills	41 (27.0%)	27.0**%**	83 (24.5%)	24.5**%**	0.557
Cough	64 (42.1%)	42.1**%**	166 (49.0%)	49.0**%**	0.159
Dyspnea	100 (65.8%)	65.8**%**	214 (63.1%)	63.1**%**	0.570
Desaturation	96 (63.2%)	63.2**%**	195 (57.5%)	57.5**%**	0.240
Diarrhea	29 (19.1%)	19.1**%**	72 (21.2%)	21.2**%**	0.584
Abdominal Pain	13 (8.6%)	8.6**%**	20 (5.9%)	5.9**%**	0.278
Nausea/Vomiting	14 (9.2%)	9.2**%**	30 (8.8%)	8.8**%**	0.897
Myalgia	49 (32.2%)	32.2**%**	99 (29.2%)	29.2**%**	0.498
Chest Pain	6 (3.9%)	3.9**%**	31 (9.1%)	9.1**%**	0.044
Sore Throat	3 (2.0%)	2.0**%**	23 (6.8%)	6.8**%**	0.028

The rates of comorbid medical conditions were significantly higher in patients with T2DM than in those without T2DM. Hypertension (76.35%), dyslipidaemia (58.6%) and coronary artery disease (CAD) (33.6%) were the most common comorbidities reported among the patients with T2DM (p < 0.001). The rates of other comorbidities, including heart failure, obstructive lung disease, chronic kidney disease and cancer of any type, were not significantly different between the two groups.

Chest X-ray and CT findings at admission were similar between the patients with and without T2DM. Albeit not statistically significant, the rate of pleural effusion on CT scan was higher in patients with T2DM than in those without T2DM (17.5% vs. 11.2%, p = 0.07). Finally, the rates of Chest X-ray consolidation and ground glass opacities on CT scans were comparable between the two groups.

As shown in **Table 3**, the comparison of the laboratory results at admission revealed no significant differences in white blood cell count, neutrophil and lymphocyte percentages and C-reactive protein, D-dimer, lactic acid and interleukin (IL)-6 levels between the patients with and without T2DM. Nevertheless, the two groups exhibited significant differences in the levels of creatinine and the cardiac enzymes troponin T and creatine kinase-MB. Specifically, 30.4% and 15.0% of the patients with and without T2DM had creatinine levels equal to or greater than 1.17 mg/L (p < 0.001), respectively. Furthermore, 93.3% and 83.1% of the patients with and without T2DM had troponin T levels equal to or greater than 4 ng/dL (p = 0.004).

**Table 3:** Laboratory findings of diabetic and non-diabetic patients admitted for COVID-19 infection.

	Type 2 Diabetes		No Type 2 Diabetes		
	N (%)		N (%)		*p*-value
**Laboratory results**					
***White Blood Cell count (in 10***^***9***^ ***cells/L)***					
< 5.2	56 (37.1%)	37.1**%**	107 (31.8%)	31.8**%**	
5.2–12.4	79 (52.3%)	52.3**%**	201 (59.8%)	59.8**%**	
> 12.4	16 (10.6%)	10.6**%**	28 (8.3%)	8.3**%**	0.291
***Neutrophil percentage (%)***					
<40%	5 (3.3%)	3.3**%**	6 (1.8%)	1.8**%**	
40%– 74%	41 (27. 2%)	27.2**%**	79 (23.5%)	23.5**%**	
>74%	105 (69.5%)	69.5**%**	251 (74.7%)	74.7**%**	0.365
***Lymphocyte percentage (%)***					
<19%	125 (82.8%)	82.8**%**	267 (79.5%)	79.5**%**	
19%– 48%	25 (16.6%)	16.6**%**	63 (18.8%)	18.8**%**	
>48%	1 (0.7%)	0.7**%**	6 (1.8%)	1.8**%**	0.513
***C-Reactive Protein levels (mg/L)***					
<0.7	9 (6.6%)	6.6**%**	13 (4.2%)	4.2**%**	
≥0.7	127 (93.4%)	93.4**%**	299 (95.8%)	95.8**%**	0.270
***Creatinine levels (mg/L)***					
≤1.17	103 (69.6%)	69.6**%**	284 (85.0%)	85.0**%**	
>1.17	45 (30.4%)	30.4**%**	50 (15.0%)	15.0**%**	<0.001
***D-dimer levels (mcg/mL)***					
<0.5	33 (24.3%)	24.3**%**	77 (25.8%)	25.8**%**	
≥0.5	103 (75.7%)	75.7**%**	221 (74.2%)	74.2**%**	0.727
***Troponin-T (ng/dL)***					
≤4	9 (6.7%)	6.7**%**	46 (16.9%)	16.9**%**	
>4	126 (93.3%)	93.3**%**	226 (83.1%)	83.1**%**	0.004
***CK-MB (IU/L)***					
≤25	127 (97.7%)	97.7**%**	261 (100.0%)	100.0**%**	
>25	3 (2.3%)	2.3**%**	0 (0.0%)	0.0**%**	0.014
***Lactic acid (mmol/L)***					
≤2.20	56 (63.6%)	63.6**%**	93 (69.9%)	69.9**%**	
>2.20	32 (36.4%)	36.4**%**	40 (30.1%)	30.1**%**	0.329
***IL-6 (pg/mL)***					
<7	6 (5.1%)	5.1**%**	20 (7.8%)	7.8**%**	
7–39.99	40 (33.9%)	33.9**%**	99 (38.7%)	38.7**%**	
≥40	72 (61.0%)	61.0**%**	137 (53.5%)	53.5**%**	0.337

**Table 4** summarises the comparison of length of hospitalisation, oxygen therapy, ICU course, length of ICU stays and mortality rate between the patients with and without T2DM. The length of hospitalisation and the rate of oxygen therapy were not significantly different between the two groups. The patients with T2DM were more likely to be admitted to the ICU (34.2%, p = 0.005), require invasive ventilation (18.4%, p = 0.013) and receive vasopressors (16.4%, p = 0.043) compared with those without T2DM. However, the patients with T2DM were not more likely to be admitted to the ICU at presentation. The mortality rate was 14.9% in the T2DM group and 11.7% in the non-T2DM group (p = 0.337).

**Table 4:** Clinical outcome of diabetic and non-diabetic patients admitted for COVID-19 infection.

	Type 2 Diabetes		No Type 2 Diabetes		
	N (%)		N (%)		*p*-value
**Length of Hospitalization**					
5 days or less	50 (32.9%)		128 (37.8%)		
5 to 10 days	51 (33.6%)		112(33.0%)		
10 to 15 days	21 (13.8%)		35 (10.3%)		
15 to 20 days	11 (7.2%)		28 (8.3%)		
More than 20 days	19 (12.5%)		36 (10.6%)		0.684
**Oxygen therapy**					
Received Oxygen Therapy	103 (67.8%)		221 (65.2%)		0.578
**ICU stay**					
Total ICU stay	52 (34.2%)		75 (22.1%)		0.005
Originally admitted to ICU	32 (61.5%)		39 (52.0%)		0.287
Use of Invasive Ventilation	28 (18.4%)		35 (10.3%)		0.013
Use of Vasopressors	25 (16.4%)		34 (10.0%)		0.043
**ICU Length of Stay**					
5 days or less	16 (30.8%)		29 (38.7%)		
5 to 10 days	13 (25.0%)		10 (13.3%)		
10 to 15 days	10 (19.2%)		17 (22.7%)		
15 to 20 days	5 (9.6%)		9 (12.0%)		
More than 20 days	8 (15.4%)		10 (13.3%)		0.514
**Status on discharge**					
Improved and discharged to quarantine or cured	126 (85.1%)		294 (88.3%)		
Deceased	22 (14.9%)	14.9**%**	39 (11.7%)		0.337

**Table 5** shows the results of the multivariable logistic regression analysis with the hospitalisation outcomes of mortality and improvement/cure as dependent variables. The regression analysis yielded five independent variables that were significantly associated with the odds of mortality, with sex maintained as a controlled covariate. The odds of mortality were lower in patients with T2DM than in those without T2DM (OR, 0.27; 95% CI, 0.08–0.93). Conversely, age was associated with increased odds of mortality, with an 8% increment per year (OR, 1.08; 95% CI, 1.03–1.13). Longer ICU stay was associated with decreased odds of mortality (OR, 0.38; 95% CI, 0.33–0.95), whereas vasopressor use and invasive ventilation were associated with increased odds of mortality (OR, 19.83; 95% CI, 4.36–90.20 and OR, 9.95; 95% CI, 2.24–44.32, respectively).

**Table 5:** Logistic regression for hospitalization outcome (deceased vs improved/cured).

	OR	95% CI of OR	p-value
**Type 2 DM**	0.27	(0.08–0.93)	.039
**Age** (continuous)	1.08	(1.03–1.13)	.001
**Male**	0.38	(0.10–1.47)	.160
**ICU LOS** (continuous)	0.56	(0.33–0.95)	.031
**Use of Vasopressor**	19.83	(4.36–90.20)	< .001
**Use of Invasive Ventilation**	9.95	(2.24–44.32)	.003

## Discussion

In this retrospective observational study, patients with T2DM and COVID-19 were older than their non-diabetic counterparts, while both cohorts were predominantly males. Patients with T2DM presented with lower temperatures and milder symptoms. HTN, DL and CAD were more prevalent in patients with TD2M compared with non-diabetic patients. Patients with T2DM had higher levels of creatinine, Troponin-T, and creatine kinase-MB. The patients with T2DM were more likely to be admitted to the ICU, require invasive ventilation, and receive vasopressors, while length of hospitalisation and oxygen therapy rates were not significantly different between both groups. A multivariable logistic regression analysis showed that patients with T2DM had lower odds of mortality. The regression analysis also showed that age, vasopressor use and invasive ventilation were associated with increased odds of mortlity while increase ICU stay was associated with decreased mortality odds.

T2DM tends to more common in older individuals, and age has been determined to be an important contributor to the hospital course and clinical outcomes in patients infected with SARS-CoV-2 [17]. Studies have shown that patients with T2DM admitted for COVID-19 are significantly older [18], consistent with our findings. Studies have also shown that males are more likely to be infected with SARS-CoV-2 and to be admitted for COVID-19 [17–20], which might be due to the higher risk of exposure, as men were found to report less fear and negative perceptions of health consequences of COVID-19 than women, while also showing less responsibility for preventive measures [21,22]. In the present study, the patients admitted to the hospital due to COVID-19 were predominantly male in the overall cohort, with no significant sex difference between the patients with and without T2DM. Additionally, the smoking status did not differ between the two groups.

One study showed that the most common symptoms of COVID-19 in patients with T2DM are fever, cough and dyspnoea [9]. The present study supported these findings. Some signs and symptoms tend to be predominantly observed in patients with T2DM infected with SARS-CoV-2. A multi-centre study in China reported that fever (87.04%), dry cough (66%) and expectoration (39.62%) were the most common presenting symptoms [23]. The patients with T2DM were less likely to experience high-grade fever > 39.3°C (83.9% vs. 69.9%, p = 0.005) and chest pain (3.9% vs. 9.1%, p = 0.044) compared with the patients without T2DM. In the present study, we also found that the patients with T2DM were less likely to experience sore throat and chest pain. The lower rates of physical pain and discomfort might be attributed to the neuropathic and micro/microvascular complications of DM. For instance, patients with DM are half as likely to experience chest pain during acute coronary syndrome [24]. These differences in clinical presentation between the patients with and without T2DM are crucial and require a high index of suspicion during the assessment of patients with T2DM. The lack of flagrant symptoms might conceal the apparent severity of the infection and affect appropriate management, in turn impacting the clinical course and outcomes in patients with T2DM. One theory posits that patients with DM may have milder symptoms due to compromised inflammatory response. Studies have demonstrated lower activity of natural killer cells in patients with DM, leading to impaired immune function and higher susceptibility to infections [25]. Additionally, high glucose levels induce the production of transforming growth factor β1, which suppresses the immune system by inhibiting a variety of cytokines, including IL-2, IL-6 and IL-10 [26]. Studies in mice have shown decreased expression of intracellular adhesion molecules and certain cytokines, such as C-X-C motif chemokine ligand 1 and 2, IL-1β and TNF-α, in the diabetic state, which might lead to decreased lymphocyte recruitment that may explain the weaker immune response, milder symptoms and worse outcomes with Sars-CoV-2 infection [27,28].

Patients with DM tend to have several comorbidities. DM causes hypertension through atherosclerosis and the formation of atherosclerotic plaques in blood vessels. DM also increases the risk of CAD through deposition of glycosylation end-products in coronary vessels endothelial cells. Dyslipidaemia is also a common comorbidity of DM due to several interrelated biochemical mechanisms [29]. In a previous study, hypertension (50.0%), CAD (27.1%) and cerebrovascular disease (10.4%) were more prevalent in in patients with DM admitted for severe COVID-19 [30]. Indeed, in the present study cohort, hypertension, dyslipidaemia and CAD were the most common comorbidities with higher frequency in the patients with T2DM compared with those without T2DM.

The radiological findings did not exhibit a significant difference between the patients with and without T2DM; however, the rate of pleural effusion was significantly higher in patients with T2DM than in those without T2DM, which might be attributable to the higher prevalence of CAD in patients with T2DM. In addition, DM is known to cause diabetic nephropathy through structural and functional damage as well as other comorbidities such as hypertension [31], which might also explain the significantly higher levels of creatinine observed in patients with T2DM and COVID-19 in the present study.

Elevated levels of troponin (31%) in patients with COVID-19 were reported in several studies [32–35] that highlighted the association of high troponin levels with higher mortality rates [36]. Manocha and colleagues found the troponin to be the only independent predictor of 30-day mortality [34]. Another study reported that higher troponin levels were associated with worse clinical outcomes [32]. In the present study, the significantly higher Troponin-T levels in patients with T2DM might explain the worse clinical outcomes in these patients in comparison with those without T2DM. However, troponin does not always indicate a myocardial injury and can be elevated in severe respiratory infection, hypoxia, sepsis, systemic inflammation, pulmonary thrombosis, cytokine storm and myocarditis [37]. The underlying cause of troponin elevation in COVID-19 remains unclear. However, the most prominent speculation involves endothelial dysfunction and the thrombotic state, especially in the pulmonary vasculature, as well as generalised inflammation rather than actual myocardial ischaemic injury [37]. A study showed that elevated levels of high-sensitivity cardiac troponin T and high-sensitivity cardiac troponin-I reflected the health status of elderly patients with DM and were associated with heart failure, CAD, chronic kidney disease and severe hypoglycaemia [38]. Segre et al. found that patients with DM and CAD had higher levels of troponin than those without CAD (12.0 vs. 7.0 pg/mL) and that the troponin levels varied depending on the patients’ medications [37,38]. In the current study, creatine kinase-MB was elevated in only three patients in the T2DM group and none in the non-T2DM group suggesting that the combined effect of DM and COVID-19 might have led to increased troponin T levels even in the absence of ischaemic injury. Troponin is an inflammatory biomarker and might be considered to be a tool to evaluate patients with DM and COVID-19, especially considering that these patients might be less symptomatic while being prone to worse clinical course compared with patients without DM. In patients with DM, troponin might be a helpful prognostic marker to predict progression of infection with related cytokine storm and the hypercoagulable status [39,40].

Accumulating evidence has shown that patients with T2DM are more likely to be hospitalised and to experience worse outcomes and higher rates of mortality from infections compared with patients without T2DM [6,41]. An elevated inflammatory state by certain macrophages might be a contributing factor to increased tissue damage, leading to nephropathy, neuropathy, retinopathy and cardiovascular diseases in patients with T2DM [42]. The prevalence of DM is higher in patients severely affected by COVID-19, with one study reporting that 57.14% of patients with severe COVID-19 had T2DM [19]. Patients with DM are not only more likely to contract SARS-CoV-2 but also to experience worse clinical outcomes, making them more likely to be admitted to the ICU and receive mechanical ventilation [8]. The present study corroborates these findings.

The current study also confirms the expected increase in mortality risk with patient age in both the patients with and without T2DM. The inherent older age of patients with T2DM at presentation for COVID-19 should therefore be regarded as an important factor underlying adverse outcomes observed in these patients, in addition to T2DM itself [43]. Our analyses also indicated that patients receiving invasive mechanical ventilation and vasopressors were at a higher likelihood of mortality, possibly because of the already high risk of mortality and clinical deterioration. Although patients with DM might have fewer symptoms and similar C-reactive protein and IL-6 levels when compared with those without DM, they are still at higher risk of worse clinical course and increased need for ICU care. Thus, continuous monitoring and aggressive management must be provided for patients with DM. Interestingly, there were no significant differences in the length of hospitalisation, ICU stays and need for oxygen therapy between the patients with and without T2DM. These findings further support the notion that if adequately managed, patients with T2DM and COVID-19 may not require longer treatment compared with those without T2DM. Of note, although a longer ICU stay was associated with lower odds of mortality in the present study, this finding was most likely a natural effect of longer ICU stays, which eventually lead to better prognosis compared with shorter ICU stays that reflect patient’s demise. Finally, as well as unlike most of the previous studies, we did not find a statistically significant disparity in mortality between the patients with and without T2DM hospitalised for COVID-19. In fact, our regression analysis indicated that the odds of mortality were lower in patients with T2DM than in those without T2DM. Multiple factors might have played into these findings: Patients with T2DM infected by COVID-19 who present with respiratory distress can usually attribute this presentation to acute respiratory distress syndrome(ARDS) or newly-induced congestive heart failure (CHF) related to their T2DM as shown in various studies; Nichols et al. found that patients with diabetes were much more likely to develop CHF than patients without diabetes with younger age-groups demonstrating the biggest such difference [44]. If managed properly, these patients would potentially recover faster than their counterparts without T2DM who present with pulmonary complications most likely caused by intrinsic lung disease that make management and recovery more difficult, thus increasing their mortality rates. Moreover, statin use in patients with COVID-19 has been shown to decrease mortality, and one meta-analysis of retrospective observational studies found the decrease to be up to 35% [45]. Since many patients with T2DM have comorbidities such as HTN and dyslipidaemia, statin administration in this cohort of patients might also play a role in decrease mortality rates compared with patients without T2DM as seen in the present study. In addition, as mentioned previously, the impaired immune response seen in patients with T2DM might prevent the rise of a potentially deleterious cytokine surge in response to COVID-19 infection and inadvertently decrease mortality rates in these patients. It is also possible that patients with T2DM presented with more risk factors for COVID-19 related mortality, and therefore more aggressive management was targeted towards this group contributing to their lower odds of mortality seen. Despite a more severe in-hospital clinical course and higher rates of ICU admission, the patients with T2DM were not more likely to die from COVID-19 when compared with those without T2DM in the current study. These findings indicate that the ethnicities of the populations studied may also play a role in influencing outcomes in patients with T2DM and COVID-19, as this is study about Lebanese patients presenting to a single centre in Lebanon.

### Limitations

The present study has several limitations that should be acknowledged. First, this was a single-centre study with a relatively small sample size, which reduces its statistical power. However, this is the first study in Lebanon to date examining the clinical presentation and outcomes of COVID-19 in patients with T2DM in comparison with those without T2DM. Furthermore, infectious disease and ICU specialists have been continually adjusting specific treatments for COVID-19 in parallel with the emergence of new data and international guidelines during the progression of the COVID-19 pandemic. Not all patients in the present study were treated using the same guidelines, which might have confounded the evaluation of patient outcomes. The medications administered to the patients with T2DM varied depending on the specific comorbidities of the patients. Nonetheless, the number of comorbidities is expected to be higher in patients with DM and should, if any, contribute to the worse clinical outcomes observed in such patients. Lastly, the logistic regression model may have produced inflated estimates due to the small sample size and hence a prevalence rate ratio may be more suited for such data [46,47]. Nevertheless, the findings of this study, while interpreted with caution, warrant further investigation into the role of T2DM with COVID-19 in larger centres with bigger sample sizes.

### Conclusion

Similar to previous studies, we found that patients with T2DM hospitalised for COVID-19 were more likely to be older males presenting with low-grade fever and milder symptoms, have a more complicated course of illness and to present with higher specific biomarkers compared with patients without T2DM. Unlike most studies, however, these patients had lower odds of mortality, suggesting that aggressive management may lead to better outcomes. This is the first study that sheds some light on COVID-19 in Diabetic patients in the region. Nevertheless, further studies are needed to help understand the prognoses of patients with T2DM and COVID-19 and guide their management.

## Supporting information

S1 Data (XLSX)

S1 File (DOCX)

S2 File (DOCX)

## References

[1] WHO Coronavirus (COVID-19) Dashboard | WHO Coronavirus (COVID-19) Dashboard With Vaccination Data. Accessed June 29, 2022. https://covid19.who.int/.

[2] Cutler DM, Summers LH. The COVID-19 Pandemic and the $16 Trillion Virus. JAMA. 2020;324(15):1495–1496. doi: 10.1001/jama.2020.19759 33044484 PMC7604733

[3] Wu S, Xue L, Legido-Quigley H, et al. Understanding factors influencing the length of hospital stay among non-severe COVID-19 patients: A retrospective cohort study in a Fangcang shelter hospital. PloS one. 2020;15(10):e0240959–e0240959. doi: 10.1371/journal.pone.0240959 33085709 PMC7577449

[4] Singh AK, Gupta R, Ghosh A, Misra A. Diabetes in COVID-19: Prevalence, pathophysiology, prognosis and practical considerations. Diabetes Metab Syndr. 2020;14(4):303–310. doi: 10.1016/j.dsx.2020.04.004 32298981 PMC7195120

[5] Akbar DH. Bacterial pneumonia: comparison between diabetics and non-diabetics. Acta Diabetol. 2001;38(2):77–82. doi: 10.1007/s005920170017 11757805

[6] Yang JK, Feng Y, Yuan MY, et al. Plasma glucose levels and diabetes are independent predictors for mortality and morbidity in patients with SARS. Diabetic medicine. 2006;23(6):623–628. doi: 10.1111/j.1464-5491.2006.01861.x 16759303

[7] Badawi A, Ryoo SG. Prevalence of comorbidities in the Middle East respiratory syndrome coronavirus (MERS-CoV): a systematic review and meta-analysis. Int J Infect Dis. 2016;49:129–133. doi: 10.1016/j.ijid.2016.06.015 27352628 PMC7110556

[8] Izzi-Engbeaya C, Distaso W, Amin A, et al. Adverse outcomes in COVID-19 and diabetes: a retrospective cohort study from three London teaching hospitals. BMJ open diabetes research & care. 2021;9(1). doi: 10.1136/bmjdrc-2020-001858 33408084 PMC7789097

[9] Yan Y, Yang Y, Wang F, et al. Clinical characteristics and outcomes of patients with severe covid-19 with diabetes. BMJ open diabetes research & care. 2020;8(1):e001343. doi: 10.1136/bmjdrc-2020-001343 32345579 PMC7222577

[10] Wu J, Zhang J, Sun X, et al. Influence of diabetes mellitus on the severity and fatality of SARS-CoV-2 (COVID-19) infection. Diabetes Obes Metab. 2020;22(10):1907–1914. doi: 10.1111/dom.14105 32496012 PMC7300679

[11] Kim L, Garg S, O’Halloran A, et al. Risk Factors for Intensive Care Unit Admission and In-hospital Mortality Among Hospitalized Adults Identified through the US Coronavirus Disease 2019 (COVID-19)-Associated Hospitalization Surveillance Network (COVID-NET). Clin Infect Dis. 2021;72(9):e206–e214. doi: 10.1093/cid/ciaa1012 32674114 PMC7454425

[12] Al-Salameh A, Lanoix JP, Bennis Y, et al. Characteristics and outcomes of COVID-19 in hospitalized patients with and without diabetes. Diabetes Metab Res Rev. 2021;37(3):e3388. doi: 10.1002/dmrr.3388 32683744 PMC7404605

[13] Kastora S, Patel M, Carter B, Delibegovic M, Myint PK. Impact of diabetes on COVID-19 mortality and hospital outcomes from a global perspective: An umbrella systematic review and meta-analysis. Endocrinol Diabetes Metab. 2022;5(3):e00338. doi: 10.1002/edm2.338 35441801 PMC9094465

[14] Khan MAB, Hashim MJ, King JK, Govender RD, Mustafa H, Al Kaabi J. Epidemiology of Type 2 Diabetes–Global Burden of Disease and Forecasted Trends. J Epidemiol Glob Health. 2020;10(1):107–111. doi: 10.2991/jegh.k.191028.001 32175717 PMC7310804

[15] Diabetes. Accessed December 21, 2021. https://www.who.int/news-room/fact-sheets/detail/diabetes.

[16] Members. Accessed February 15, 2022. https://idf.org/our-network/regions-members/middle-east-and-north-africa/members/39-lebanon.html.

[17] Zhou W, Ye S, Wang W, Li S, Hu Q. Clinical Features of COVID-19 Patients with Diabetes and Secondary Hyperglycemia. Takayuki Masaki, Masaki T, eds. Journal of diabetes research. 2020;2020(Journal Article):3918723–3918723. doi: 10.1155/2020/3918723 33062712 PMC7545437

[18] Alshukry A, Bu Abbas M, Ali Y, et al. Clinical characteristics and outcomes of COVID-19 patients with diabetes mellitus in Kuwait. Heliyon. 2021;7(4):e06706–e06706. doi: 10.1016/j.heliyon.2021.e06706 33842709 PMC8020058

[19] Hafidh K, Abbas S, Khan A, Kazmi T, Nazir Z, Aldaham T. The Clinical Characteristics and Outcomes of COVID-19 Infections in Patients with Diabetes at a Tertiary Care Center in the UAE. Dubai Diabetes and Endocrinology Journal. 2021;26(4):158–163. doi: 10.1159/000512232

[20] Wang D, Hu B, Hu C, et al. Clinical Characteristics of 138 Hospitalized Patients With 2019 Novel Coronavirus-Infected Pneumonia in Wuhan, China. JAMA. 2020;323(11):1061–1069. doi: 10.1001/jama.2020.1585 32031570 PMC7042881

[21] Alsharawy A, Spoon R, Smith A, Ball S. Gender Differences in Fear and Risk Perception During the COVID-19 Pandemic. Frontiers in Psychology. 2021;12. Accessed November 29, 2022. doi: 10.3389/fpsyg.2021.689467 34421741 PMC8375576

[22] Bwire GM. Coronavirus: Why Men are More Vulnerable to Covid-19 Than Women? SN Compr Clin Med. 2020;2(7):874–876. doi: 10.1007/s42399-020-00341-w 32838138 PMC7271824

[23] Yi H, Lu F, Jin X, et al. Clinical characteristics and outcomes of coronavirus disease 2019 infections among diabetics: A retrospective and multicenter study in China. Journal of diabetes. 2020;12(12):919–928. doi: 10.1111/1753-0407.13098 32725691

[24] DeVon HA, Penckofer S, Larimer K. The association of diabetes and older age with the absence of chest pain during acute coronary syndromes. Western journal of nursing research. 2008;30(1):130–144. doi: 10.1177/0193945907310241 18182562 PMC2247416

[25] Kim JH, Park K, Lee SB, et al. Relationship between natural killer cell activity and glucose control in patients with type 2 diabetes and prediabetes. J Diabetes Investig. 2019;10(5):1223–1228. doi: 10.1111/jdi.13002 30618112 PMC6717814

[26] Reinhold D, Ansorge S. Elevated Glucose Levels Stimulate Transforming Growth Factor-β1 (TGF-β1), Suppress Interleukin IL-2, IL-6 and IL-10 Production and DNA Synthesis in Peripheral Blood Mononuclear Cells. Horm Metab Res. 1996;28(6):267–270. doi: 10.1055/s-2007-979789 8811326

[27] Kumar M, Roe K, Nerurkar PV, et al. Reduced immune cell infiltration and increased pro-inflammatory mediators in the brain of Type 2 diabetic mouse model infected with West Nile virus. J Neuroinflammation. 2014;11:80. doi: 10.1186/1742-2094-11-80 24750819 PMC4001407

[28] Martinez N, Ketheesan N, Martens GW, West K, Lien E, Kornfeld H. Defects in early cell recruitment contribute to the increased susceptibility to respiratory Klebsiella pneumoniae infection in diabetic mice. Microbes Infect. 2016;18(10):649–655. doi: 10.1016/j.micinf.2016.05.007 27256462 PMC10687709

[29] Kimak E bieta, Ksi ek A, Baranowicz-G szczyk I, Solski J. Disturbed Lipids, Lipoproteins and Triglyceride-Rich Lipoproteins as Well as Fasting and Nonfasting Non-High-Density Lipoprotein Cholesterol in Post-Renal Transplant Patients. Renal failure. 2007;29(6):705–712. doi: 10.1080/08860220701460111 17763166

[30] Yan Y, Yang Y, Wang F, et al. Clinical characteristics and outcomes of patients with severe covid-19 with diabetes. BMJ open diabetes research & care. 2020;8(1):e001343. doi: 10.1136/bmjdrc-2020-001343 32345579 PMC7222577

[31] Alicic RZ, Rooney MT, Tuttle KR. Diabetic Kidney Disease. Clin J Am Soc Nephrol. 2017;12(12):2032–2045. doi: 10.2215/CJN.11491116 28522654 PMC5718284

[32] Ali J, Khan FR, Ullah R, et al. Cardiac Troponin I Levels in Hospitalized COVID-19 Patients as a Predictor of Severity and Outcome: A Retrospective Cohort Study. Cureus. 2021;13(3). doi: 10.7759/cureus.14061 33898144 PMC8061753

[33] Majure DT, Gruberg L, Saba SG, et al. Usefulness of Elevated Troponin to Predict Death in Patients With COVID-19 and Myocardial Injury. Am J Cardiol. 2021;138:100–106. doi: 10.1016/j.amjcard.2020.09.060 33058800 PMC7550867

[34] Manocha KK, Kirzner J, Ying X, et al. Troponin and Other Biomarker Levels and Outcomes Among Patients Hospitalized With COVID‐19: Derivation and Validation of the HA2T2 COVID‐19 Mortality Risk Score. Journal of the American Heart Association. 2021;10(6):e018477. doi: 10.1161/JAHA.120.018477 33121304 PMC8174190

[35] Tersalvi G, Vicenzi M, Calabretta D, Biasco L, Pedrazzini G, Winterton D. Elevated Troponin in Patients With Coronavirus Disease 2019: Possible Mechanisms. J Card Fail. 2020;26(6):470–475. doi: 10.1016/j.cardfail.2020.04.009 32315733 PMC7166030

[36] Wibowo A, Pranata R, Akbar MR, Purnomowati A, Martha JW. Prognostic performance of troponin in COVID-19: A diagnostic meta-analysis and meta-regression. International Journal of Infectious Diseases. 2021;105:312–318. doi: 10.1016/j.ijid.2021.02.113 33667694 PMC7923942

[37] Imazio M, Link to external site this link will open in a new window, Klingel K, et al. COVID-19 pandemic and troponin: indirect myocardial injury, myocardial inflammation or myocarditis? Heart. 2020;106(15):1127–1131. doi: 10.1136/heartjnl-2020-317186 32499236

[38] Cardiac Troponin Levels May Reflect Health Status of Older Patients With Diabetes. Endocrinology Advisor. Published July 13, 2019. Accessed February 23, 2022. https://www.endocrinologyadvisor.com/home/topics/diabetes/ctnt-ctni-markers-for-comorbidity-burden-mortality-risk-in-diabetes/.

[39] Segre CAW, Hueb W, Garcia RMR, et al. Troponin in diabetic patients with and without chronic coronary artery disease. BMC Cardiovascular Disorders. 2015;15(1):72. doi: 10.1186/s12872-015-0051-z 26195004 PMC4508806

[40] Wega AB, Wabalo EK, Edae CK, Awgichew GB. Cardiac Troponin-I Status of Type-2 Diabetic Patients on Anti-Diabetic Drugs Treatment at Jimma Medical Center, Jimma, Southwest Ethiopia. RRCC. 2021;12:1–7. doi: 10.2147/RRCC.S313432

[41] Shah BR, Hux JE. Quantifying the Risk of Infectious Diseases for People With Diabetes. Diabetes care. 2003;26(2):510–513. doi: 10.2337/diacare.26.2.510 12547890

[42] Meshkani R, Vakili S. Tissue resident macrophages: Key players in the pathogenesis of type 2 diabetes and its complications. Clinica chimica acta. 2016;462(Journal Article):77–89. doi: 10.1016/j.cca.2016.08.015 27570063

[43] Wang D, Hu B, Hu C, et al. Clinical Characteristics of 138 Hospitalized Patients With 2019 Novel Coronavirus-Infected Pneumonia in Wuhan, China. JAMA. 2020;323(11):1061–1069. doi: 10.1001/jama.2020.1585 32031570 PMC7042881

[44] Nichols GA, Gullion CM, Koro CE, Ephross SA, Brown JB. The Incidence of Congestive Heart Failure in Type 2 Diabetes: An update. Diabetes Care. 2004;27(8):1879–1884. doi: 10.2337/diacare.27.8.1879 15277411

[45] Kollias A, Kyriakoulis KG, Kyriakoulis IG, et al. Statin use and mortality in COVID-19 patients: Updated systematic review and meta-analysis. Atherosclerosis. 2021;330:114–121. doi: 10.1016/j.atherosclerosis.2021.06.911 34243953 PMC8233054

[46] Martinez BAF, Leotti VB, Silva G de S e, Nunes LN, Machado G, Corbellini LG. Odds Ratio or Prevalence Ratio? An Overview of Reported Statistical Methods and Appropriateness of Interpretations in Cross-sectional Studies with Dichotomous Outcomes in Veterinary Medicine. Front Vet Sci. 2017;4:193. doi: 10.3389/fvets.2017.00193 29177157 PMC5686058

[47] Lee J, Chia KS. Estimation of prevalence rate ratios for cross sectional data: an example in occupational epidemiology. Br J Ind Med. 1993;50(9):861–862. doi: 10.1136/oem.50.9.861 8398881 PMC1061320

